# Molecular Hydrogen: A Promising Adjunctive Strategy for the Treatment of the COVID-19

**DOI:** 10.3389/fmed.2021.671215

**Published:** 2021-10-22

**Authors:** Yingning Li, Zhen Wang, Naqi Lian, Yuzun Wang, Weiqiang Zheng, Keliang Xie

**Affiliations:** ^1^Department of Anesthesiology, Tianjin Medical University General Hospital, Tianjin Research Institute of Anesthesiology, Tianjin, China; ^2^Department of Critical Care Medicine, Tianjin Medical University General Hospital, Tianjin, China; ^3^College of Anesthesiology, Translational Research Institute of Intensive Care Medicine, College of Anesthesiology, Weifang Medical University, Weifang, China

**Keywords:** COVID-19, molecular hydrogen, inflammation, cytokines, treatment

## Abstract

Coronavirus disease 2019 (COVID-19) is an acute respiratory disease caused by a severe acute respiratory syndrome coronavirus 2 (SARS-CoV-2), which has no specific and effective treatment. The pathophysiological process of the COVID-19 is an excessive inflammatory response after an organism infects with a virus. Inflammatory storms play an important role in the development of the COVID-19. A large number of studies have confirmed that hydrogen has a therapeutic effect on many diseases *via* inhibiting excessive inflammatory cells and factors. Recently, a study led by the Academician Zhong Nanshan in China on the treatment of the patients with the COVID-19 by inhalation of a mixed gas composed of hydrogen and oxygen has attracted widespread international attention and hydrogen therapy has also been included in a new treatment plan for the COVID-19 in China. This study mainly describes the mechanism of occurrence of the COVID-19, summarizes the therapeutic effects and underlying mechanisms of hydrogen on the critical disease, and analyzes the feasibility and potential therapeutic targets of hydrogen for the treatment of the COVID-19.

## Characteristics And Related Mechanisms Of The Covid-19

Coronavirus disease 2019 (COVID-19) is an acute respiratory disease caused by a novel virus called severe acute respiratory syndrome coronavirus 2 (SARS-CoV-2). By the end of August 2021, the number of cases of infection and death has increased to 218 million and 4 million, respectively^1^. COVID-19 is primarily transmitted *via* the respiratory tract and close contact, with the population generally susceptible. The WHO pointed out that the patients whose main symptoms are fever, cough, and fatigue, 80% of the patients have a good prognosis. Their radiological features are the interstitial changes in the lungs. However, about 14% of the patients have a critical illness and 5% of the patients have a severe infection combined with dyspnea and/or hypoxemia followed by the rapid progression to acute respiratory distress syndrome, septic shock, and multiple organ ^1^ failure. Patients with severe illness usually require admission to an intensive care unit (ICU) for the treatment with a mortality rate of over 50%. So far, there is little evidence that any drug is effective in treating COVID-19. The current treatment mainly includes symptomatic supportive care, the application of antiviral drugs, and immunotherapy.

Severe acute respiratory syndrome coronavirus 2 is a single positive-stranded RNA virus that enters the respiratory epithelial cells by binding to the angiotensin-converting enzyme 2 (ACE2) receptor on the surface of the tissue cells *via* the S protein on the envelope surface (1–3). After entering the cell, it releases its own RNA, associates with ribosomes for the translation process, and uses the material of the host cell to synthesize its own structural proteins, functional proteins for nucleic acid replication, and viral nucleic acids. Enough structural proteins and viral genomic ribonucleic acid combine to form a progeny virus and then the vesicles are released outside the cell to continue infecting the other cells (4, 5). After the virus breaks through the first barrier, the innate immune response of the host is activated. Subsequently, the macrophages recognize the pathogen-associated molecular patterns (PAMPs) of the virus through the pattern recognition receptors on their surface and then phagocytose the virus. Meanwhile, the activated macrophages produce the pro-inflammatory factors such as tumor necrosis factor-α (TNF-α) and interleukin-1 (IL-1), which act on the vascular endothelial cells to increase the expression of the adhesion molecules and activate the chemokines. Under the action of the chemokines, the inflammatory cells migrate to the inflammatory focus to cause inflammation. Infected cells also synthesize and secrete interferons that inhibit viral replication. Cytokines released outside the cells recruit and activate the more immune cells to participate in the “antivirus war.” Immune cells will also continue to secrete the cytokines, guiding the more immune cells to the focus, forming a positive feedback regulation. If the immune system beats the virus, the inflammatory response will gradually subside and the body will recover. Once this positive feedback regulation is out of control, the immune cells of the body will be massively activated to secrete more cytokines, causing an uncontrolled inflammatory response and destroying the own structure of the body.

Autopsy of the deceased cases revealed that the lung tissues were congested, edematous, and enlarged in size with various degrees of consolidation (6). The consolidated areas were mainly the diffuse alveolar injury and exudative alveolar inflammation. There are seriflux, fibrin, and hyaline membranes in the alveolar cavity. The exudative cells are predominantly the monocytes and macrophages as well as the abundant mucous secretions in the distal bronchioles and alveoli of the respiratory tract (7). Mucus plugs of the respiratory tract leads to ventilatory dysfunction and hypoxemia. The number of the macrophages in lung tissue of the patients with the COVID-19 increased significantly, including IL-6, IL-18, interferon-γ (IFN-γ), IL-15, TNF-α, IL-1α, IL-1β, and IL-2, which potentially contribute to a “cytokine storm.” Subsequently, the cytokine storm is positively correlated with the severity of the disease (8, 9).

Simultaneously, Erlich et al. found that the excessive activation of the immune cells and persistent inflammation caused by the viral infection generate large amounts of reactive oxygen species (ROS) (10). Pro-inflammatory mediators increase the production of ROS in the mitochondria and immune cells by activating nicotinamide adenine dinucleotide phosphate (NADPH) oxidase (11). After the virus infects the body, its replication depends on the energy metabolism of the host cells and the glycolytic pathway of the host cells is significantly enhanced, resulting in the production of a large number of ROS (12). In the course of the COVID-19, the direct injury caused by the virus and ROS produced by the above two pathways leads to diffuse alveolar damage, which limits the efficiency of the alveolar gas exchange and leads to dyspnea and hypoxemia. Therefore, the lung is more prone to secondary infection (13). Therefore, preventing the virus from binding to the receptors, inhibiting the development of uncontrolled inflammation *in vivo*, and reducing cell damage caused by the products of inflammatory response can become the potential therapeutic targets of the COVID-19. Inflammatory storm and ROS are both the targets of hydrogen therapy.

## Clinical Application And Characteristics Of Hydrogen

Hydrogen, the lowest density gas known in the world, has the smallest molecular mass and some degree of reducibility. Biological research on hydrogen began in 1975 using 97.5% hydrogen and 2.5% oxygen mixture to treat the UV radiation-induced squamous cell carcinoma of mouse skin (14). In 2001, the French scholars used high-pressure hydrogen (eight standard atmospheric pressure) to treat the liver parasitic diseases, demonstrating for the first time that hydrogen has anti-inflammatory effects and proposed that the direct reaction of hydrogen with hydroxyl radical (•OH) is the molecular basis for its treatment of inflammatory damage (15). Until 2007, the Japanese scholars reported that inhalation of 2% hydrogen gas in the animals could effectively eliminate the toxic free radicals and significantly ameliorate cerebral ischemia-reperfusion injury (16), which attracted extensive international attention. Hydrogen gas has begun to become a research hotspot in the biomedical field. Hydrogen has been found to have therapeutic effects on various diseases such as tumors, sepsis, organ injury, and ischemia-reperfusion injury (17–19, 59).

There are many ways to use hydrogen such as inhalation of hydrogen, drinking hydrogen-rich water (HRW), injection of HRW, bathing with HRW, and eye drops containing dissolved H_2_. Initially, the hydrogen element used in the clinical trials was mainly in a non-gaseous form. Clinical studies have shown that drinking HRW is safe and well-tolerated and HRW containing 0.8 or 5 mM dissolved H_2_ improves the clinical symptoms in the patients with Parkinson's disease (20–22). HRW containing 7 ppm H_2_ (3.5 mg H_2_ in 500 ml water) could protect the vascular endothelium from ROS (23). Healthy adults drink 4 weeks of HRW at 1.5 L per week, which can reduce cell death and inflammation by regulating the Toll-like receptor-nuclear factor-kappa B (TLR-NF-κB) signaling and enhance the antioxidant capacity of the body. When measured by using the dissolved H_2_ analyzer, the hydrogen concentration of HRW was 0.753 ± 0.012 mg/l (24). A hydrogen-rich saline injection containing 1 ppm H_2_ can safely and effectively reduce the active phase of rheumatoid arthritis (25). Frequent use of hydrogen-rich tablets can effectively treat soft-tissue injuries in male occupational athletes (26). Oral hydrogen-rich capsules made of a blend of the hydrogen-generating minerals (46 mg of calcium and 40 mg of magnesium) can improve the insulin resistance in obese patients and H_2_ can be produced in the intestine by the following reactions: Mg + 2H_2_O → Mg(OH)_2_ + H_2_ supplying 6 ppm of H_2_ per day (27).

Hydrogen can be quickly absorbed and utilized; thus, it is more suitable for emergency patients because oral administration in emergency patients always limits liquid. Therefore, inhalation of hydrogen gas is the best option for the combination of safety and feasibility. In recent years, clinical trials have also confirmed the therapeutic effects of the inhalation of hydrogen gas. Patients with end-stage colorectal cancer treated simultaneously with 68% hydrogen and 32% oxygen were found to have an increased ratio of programmed cell death-1 (PD-1)-/CD8+ T cells in the peripheral blood, significantly longer progression-free survival, and improved prognosis (60). In addition, 3% concentration of hydrogen inhalation for the treatment of the patients with acute cerebral infarction found that the vital indications of the patients were not significantly different from those of the control group, the oxygen saturation was higher, and the degree and scope of brain injury were smaller, which could achieve the therapeutic effects after the optimal clinical therapeutic window (28). In addition, several clinical trials have confirmed that hydrogen inhalation has a positive implication on the reduction of the adverse events in the progression and treatment of postcardiac arrest syndrome after the acute myocardial infarction and non-small cell lung cancer as well as on ventricular remodeling (29–32). With respect to viral diseases, there is no evidence that hydrogen can directly act on the virus, which needs more research. Currently, the treatment with the COVID-19 consists of inhalation of a mixture of the hydrogen and oxygen (66% hydrogen; 33% oxygen) at 6 L/min *via* nasal cannula by using the Hydrogen/Oxygen Generator (model AMS-H-03, Shanghai Asclepius Meditec Co., Ltd., China). H_2_-O_2_ inhalation for 7.7 h on the basis of standard of care significantly improved the severity of the disease on day 2, including dyspnea scale, chest distress, chest pain, cough scale, and resting oxygen saturation, compared with the control group of the patients who received daily standard of care with oxygen therapy. This may be related to the reduction of inhalation resistance by hydrogen/oxygen mixture (66). Nevertheless, the trial still had some limitations, namely there was no random allocation of the patients, which may cause selection bias due to the emergency situation and, in addition, no further study of the underlying mechanism was conducted. Hydrogen inhalation reducing inhalation resistance was also demonstrated in the patients with acute severe tracheal stenosis (33). Moreover, there was a multicenter, randomized, double-blind, and parallel group controlled trial showing that inhalation of a hydrogen/oxygen mixture can significantly improve the acute exacerbation of the chronic obstructive pulmonary disease symptoms, including dyspnea, cough, and expectoration, compared with oxygen, with acceptable safety and tolerability profile (34).

## Potential Targets Of Hydrogen For The Treatment Of The Covid-19

### Neutrophils

Neutrophils, as the first defense part of the innate immunity, are considered to play a protective role upon the bacterial or fungal infection. They kill the bacteria or fungi through phagocytosis and neutrophil extracellular traps (NETs) (35). However, their role in viral infection is unclear. An autopsy of the patients who died of the COVID-19, the neutrophils infiltrated the pulmonary capillaries and alveolar cavities. The lung tissue showed acute capillaritis with fibrin deposition and mucositis with neutrophil infiltration, which was associated with the pathogenesis of the lung injury (36, 64). Transcriptome sequencing analysis of the SARS-CoV-2-infected cells showed that the infected cells expressed the neutrophil chemokines. Transcriptome sequencing analysis of bronchoalveolar lavage fluid cells of the patients also revealed upregulation of the neutrophil genes and chemokines such as TNF receptor (TNFR), IL-8, CXCR1, and CXCR2 (37). Since neutrophils are not the main inflammatory cells in the viral infection, their appearance undoubtedly aggravates inflammatory damage in the lung tissue. In the patients with the COVID-19, neutrophilia tended to predict a poor prognosis (63) and an increased neutrophil-to-lymphocyte ratio was an independent risk factor for severe disease (38). Tomar et al. found that increased mortality in patients with diabetes and cardiovascular disease was also associated with neutrophilia (39). It is found that inhalation of hydrogen gas can reduce the infiltration of the neutrophils in lung tissue, so as to alleviate inflammatory damage to the lung tissue in the disease states. Xie et al. treated mice with severe sepsis by inhalation of hydrogen gas and found that after inhalation of hydrogen gas, lung structural damage caused by inflammatory cell infiltration was significantly improved and neutrophil infiltration in the lung interstitium and alveolar space was reduced, thereby improving the survival rate of the severe septic mice modeled with cecal ligation and perforation (CLP; (58)). In the rat model of the hemorrhagic shock and resuscitation, it was found that the lung tissue myeloperoxidase (MPO) activity was lower in the 2% hydrogen inhalation group compared than in the control group and the levels of inflammatory initiation cytokine, TNF-α, and IL-1β were also reduced. Briefly, the inhalation of 2% hydrogen gas after the hemorrhagic shock and resuscitation reduced MPO activity and suppressed the pro-inflammatory mediators by reducing the infiltration of the inflammatory cells into lung tissue, thereby minimizing the degree of lung injury (40). Therefore, we hypothesized that the neutrophils could be a target for the COVID-19 hydrogen therapy.

### Macrophages

Macrophages phagocytose the damaged cells and pathogens in inflammation within the body by releasing the chemokines, leukotrienes, and prostaglandins that increase the vascular permeability and attract more inflammatory cells (41). They present the antigens to activate the adaptive immune responses. We performed single-cell RNA sequencing of the immune cells from bronchoalveolar lavage fluid of the patients with the severe COVID-19 and found that they were enriched in the pro-inflammatory monocyte-derived macrophages (42). Therefore, inhibiting excessive activation of the macrophages may be an effective way to attenuate inflammatory injury. Chen HG et al. cocultured RAW264.7 macrophages in hydrogen-rich medium with 1 μg/ml lipopolysaccharide (LPS) to obtain a sepsis cell model. The results showed that the hydrogen treatment increased the activity of heme oxygenase-1 (HO-1) in the macrophages compared with the control group and reduced the levels of pro-inflammatory factors [TNF-α, IL-1β, and high mobility group box 1 (HMGB1)] stimulated by LPS in a concentration-dependent manner, increasing levels of the anti-inflammatory factor IL-10, and decreasing levels of cellular inflammation (43). Wang et al. found that LPS induced an increase in human umbilical vein endothelial cell (HUVEC) adhesion to the monocytes, an increase in vascular cell adhesion molecule-1 (VCAM-1) and E-selectin release, and a decrease in the expression of vascular endothelial cadherin (VE-cadherin). However, hydrogen-rich fluid coculture can reduce the release of the adhesion molecules and the changes in endothelial permeability caused by LPS and prevent further development of the inflammatory responses (44). Hydrogen reduces monocyte adsorption by the endothelial adhesion molecules under inflammatory response, thus preventing the blood-borne monocytes from passing through vascular endothelium and activating into the macrophages, resulting in excessive inflammatory damage. In ovalbumin-induced asthma model of mice, inhalation of hydrogen could reverse the phagocytic defect of the macrophages in the asthmatic mice *via* nuclear factor-erythroid 2-related factor 2 (Nrf2) pathway and significantly reduced ovalbumin-induced airway hyperreactivity and inhibited inflammation and goblet cell proliferation (45). It can be seen that hydrogen can stabilize the function of the macrophages and avoid damage to the body caused by excessive activation and phagocytic defects.

### Cytokines

Cytokines play an important role in regulating the inflammatory cells by binding to the specific receptors on the target cells. Chemical mediators released by the inflammatory cells can cause vasodilation, increased permeability, and leukocyte exudation and play an important role in the initiation and progression of inflammation. Current studies suggest that the COVID-19 has a pathophysiological process similar to sepsis, i.e., the immune pathogenesis and microcirculatory dysfunction caused by systemic inflammatory cytokine storm (46). Wilson et al. found that in the serum of the patients with the severe COVID-19 or sepsis, the levels of five cytokines related to “cytokine storm” were as follows: IL-1β, IL-1RA, IL-6, IL-8, and TNF-α and there were no significant differences (47). The protective effect of hydrogen on organ injury in sepsis has been demonstrated in a variety of animal models. Xie et al. found that 2% hydrogen inhalation had therapeutic effects on acute lung injury (ALI) caused by a systemic inflammatory response model induced by intraperitoneal injection of zymosan. Hydrogen can reduce the levels of the early inflammatory factor TNF-α and the late inflammatory factor HMGB1 in the serum and lung tissue, alleviate lung tissue damage, and improve the survival rate of mice (62). The main mechanism is that hydrogen inhibits the expression of HMGB1 and alleviates tissue damage by upregulating Nrf2-mediated HO-1 pathway (48, 61). Wang et al. found that the levels of monocyte chemoattractant protein-1 (MCP-1), IL-4, and IL-6 in peripheral blood decreased significantly after inhalation of 2.4% hydrogen gas *via* a nasal catheter for 45 min in the patients with chronic obstructive pulmonary disease. Hydrogen reduces airway inflammation by reducing cytokine levels (65). According to the above studies, the use of hydrogen gas can reduce the destructive cytokine storm and lung injury caused by SARS-CoV-2 in the early stage of the COVID-19, stimulate sputum drainage, and ultimately reduce the incidence of severe disease (49).

### Reactive Oxygen Species

Reactive oxygen species are a collective term describing the chemicals formed upon by the incomplete reduction of oxygen, derived from the molecular oxygen, and formed by the redox reactions or electronic excitation including non-free radical and free radical (at least one free electron) species (50) such as superoxide anion (${\text{O}}_{2}^{-}$), hydrogen peroxide (H_2_O_2_), and •OH (51). The elevated formation of different ROS leads to molecular damage denoted as “oxidative distress.” Excess ROS can directly or indirectly destroy DNA and proteins and induce gene mutations, which are considered to be related to the development of many diseases. Despite their cytotoxic effects, •OH and H_2_O_2_ play important physiological roles at the low concentrations: they function as regulatory signaling molecules; participate in many signal transduction cascades; and regulate the biological processes such as apoptosis, cell proliferation, and differentiation (16). Similar to the other infectious diseases, large amounts of ROS are released during the COVID-19 process (13). Hydrogen is a reductive gas with selective antioxidant effects in living organisms. Ohsawa et al. first found that H_2_ has a very strong scavenging effect on •OH, a much smaller scavenging effect on nitric oxide (NO•), and a negligible scavenging effect on other reactive oxygen species such as superoxide anion radical (O_2_•) (16), which means that hydrogen can only eliminate harmful ROS while retaining other physiological ROS that plays an important role in cell signal transduction. Dong et al. found that in a CLP-induced murine sepsis model, 2% hydrogen inhalation could alleviate lung tissue damage caused by ROS and increase the oxygenation index by improving the mitochondrial function (52). However, Hancock et al. found that the direct reaction of hydrogen with the free radicals is not very active (53). Therefore, we speculate that the antioxidant effect of hydrogen in different diseases or disorders is not exactly the same. Consequently, hydrogen may work through removing toxic ROS directly and then improving the antioxidant activity of the body indirectly. In addition, ROS are also an initial signaling molecule that initiates the inflammatory response and its cascade-amplifying effects. ROS and inflammatory response can proceed in a cyclical manner, wherein ROS promotes inflammatory response and inflammation produces more ROS. This is one of the mechanisms of parenchymal tissue damage in the patient (54). Therefore, hydrogen may exert its anti-inflammatory and antioxidant effects in the COVID-19.

## Prospective

**Figure 1:**
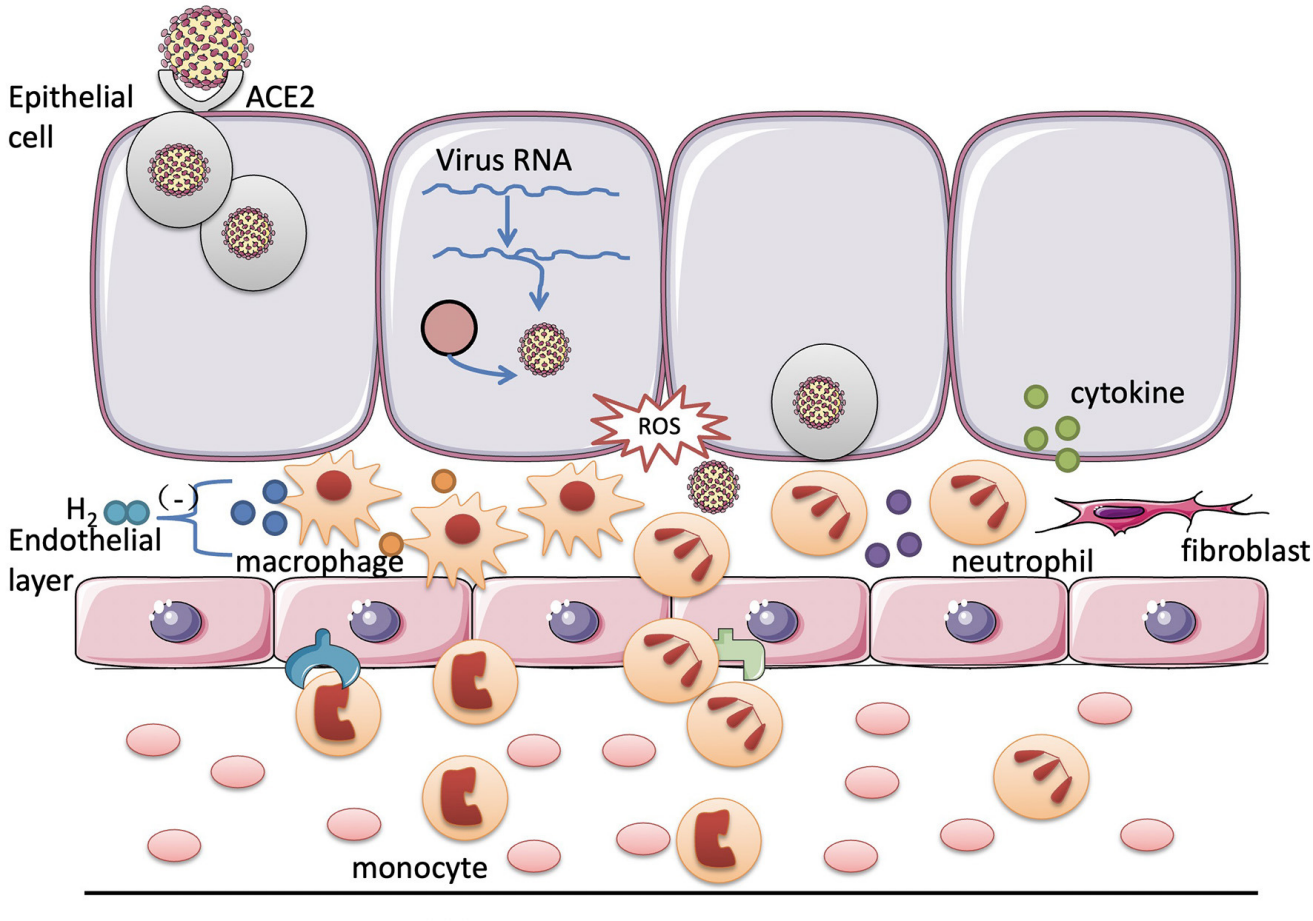
Potential targets of hydrogen for the treatment of the coronavirus disease 2019 (COVID-19). After the viral infection, the inflammatory cells in the tissues and blood are activated to destroy the virus through phagocytosis and the release of cytokines. However, excessive inflammation causes uncontrollable body damage. In the COVID-19, hydrogen may exert its protective effect on the respiratory system by inhibiting the excessive activation of the neutrophils and macrophages and reducing the release of the cytokines.

Recently, there are other reviews analyzing the possibility of hydrogen as an adjuvant treatment to the COVID-19. Russell et al. concluded that hydrogen acts on a variety of pathways to exert its anti-inflammatory and antioxidant effects in the treatment of chronic inflammatory lung diseases. Therefore, it may alleviate the severe pulmonary symptoms of the COVID-19 (54). Russell et al. found that all the domains of life have an intrinsic biological need for hydrogen from the perspective of biological evolution and hydrogen plays a therapeutic role in a variety of respiratory diseases including the COVID-19 (11). Moreover, the COVID-19 has also been reported to induce Kawasaki-like disease, which occurs in children and leads to coronary artery damage. Chen et al. considered that hydrogen can improve macrophage function and reduce myocardial ischemia-reperfusion injury *via* its anti-inflammatory effect, which may be a therapeutic target for its treatment of Kawasaki-like diseases caused by the COVID-19 (55). COVID-19 could be served as virus-induced sepsis. The main reason for the patients with COVID-19 respiratory disorders is that SARS-CoV-2 attacks the pulmonary capillary endothelial cells and triggers an immune response. Massive cellular and mucus exudate accumulation cause airway obstruction and the patients experience dyspnea. Hydrogen may inhibit tissue damage by the inflammatory cells and inflammation factors at all the stages of the inflammatory response (Figure 1). Since hydrogen can play a potential antiviral effect such as hydrogen sulfide, it remains to be further studied (56).

However, as a novel medical gas molecule, hydrogen may have the following advantages in the treatment of the patients with the COVID-19: (1) Hydrogen can directly enter the lung tissue through respiratory activities. If inhaled in combination with oxygen, oxygen can be brought into the deeper bronchus space, reducing airway resistance, increasing oxygen dispersion, and improving the respiratory function of the patient; (2) Hydrogen has a selective antioxidant effect that neutralizes the hydroxyl radicals without affecting the functional reactive oxygen. When mixed with oxygen, the potential damage from the high concentrations of oxygen can be reduced; (3) After hydrogen enters the lung tissue, it exerts anti-inflammatory effects at the multiple stages of the inflammatory response, alleviating the airway damage caused by the excessive activation of the inflammatory cells and the massive release of inflammatory factors; (4) Hydrogen can be obtained by electrolyzing water and the raw materials of the reaction are cheap and the resources are extensive. The safety of inhaling hydrogen has been demonstrated in diving medicine (67) and the treatment of the patients with the COVID-19 has also begun to show the results. Another factor must also be taken into consideration: the potential of high-concentration hydrogen to cause an explosion ignited by static electricity (57). I hope that there will be more clinical evaluations on the safety of hydrogen in the future, which will lay the foundation for the clinical application of hydrogen. Furthermore, some clinical trials have been registered on inhalational hydrogen or oral HRW for patients with the COVID-19 in the WHO clinical trials registry^2^ (Table 1). As adjunctive therapy, the mechanism of hydrogen alleviating the symptoms of the patients with the COVID-19 needs to be further clarified. At the same time, hydrogen has the potential safety concerns for the long-term treatment of diseases that needs further exploration.

**Table 1:** The current clinical trial about hydrogen therapy in patients with COVID-19.

**Study**	**Title**	**Status**	**Condition**	**Intervention**	**URL**	**Country**
1	Hydrogen-Oxygen Generator With Nebulizer in the Improvement of Symptoms in Patients Infected With COVID-19	Recruiting	COVID-19	Device: oxyhydrogen Device: Oxygen	https://ClinicalTrials.gov/show/NCT04336462	China
2	Hydrogen/Oxygen Mixed Gas Inhalation for Coronavirus Disease 2019 (COVID-19)	Completed	Covid-19 Hydrogen/Oxygen Mixed Gas Dyspnea	Device: Hydrogen Oxygen Generator with Nebulizer Other: Standard-of-care	https://ClinicalTrials.gov/show/NCT04378712	China
3	Hydrogen-oxygen Gas Mixture Inhalation in Patients With Convalescent Coronavirus Disease 2019 (COVID-19)	Not yet recruiting	Covid19 Hydrogen-oxygen Gas AMS-H-03	Device: Hydrogen-Oxygen Generator with Nebulizer, AMS-H-03 Device: OLO-1 Medical Molecular Sieve Oxygen Generator	https://ClinicalTrials.gov/show/NCT04594460	China
4	Evaluation of the Daily Intake of 0.5 L of Water Saturated With Molecular Hydrogen for 21 Days in COVID-19 Patients Treated in Ambulatory Care	Recruiting	SARS-CoV-2 Covid19 AMBULATORY CARE	Dietary Supplement: MOLECULAR HYDROGENDietary Supplement: PLACEBO MAGNESIUM	https://ClinicalTrials.gov/show/NCT04716985	France Morocco Serbia
5	Hydrogen Therapy in Patients With Moderate Covid-19	Not yet recruiting	Covid-19	Drug: Mixture 3.6% H2 in N2 (96.4%)	https://ClinicalTrials.gov/show/NCT04633980	

## Author Contributions

YL wrote the first draft of the manuscript. WZ and YW wrote the section of the manuscript. ZW and KX contributed to manuscript revision. All authors contributed to the article and approved the submitted version.

## Funding

This study was supported by a Grant from the Natural Science Foundation of Tianjin (17JCYBJC24800 to KX), the Science and Technology Support Key Program Affiliated to the Key Research and Development Plan of Tianjin Science and Technology Project (18YFZCSY00560 to KX), and the National Natural Science Foundation of China (81772043, 81971879 to KX), Beijing, China.

^2^

## Conflict of Interest

The authors declare that the research was conducted in the absence of any commercial or financial relationships that could be construed as a potential conflict of interest.

## Publisher's Note

All claims expressed in this article are solely those of the authors and do not necessarily represent those of their affiliated organizations, or those of the publisher, the editors and the reviewers. Any product that may be evaluated in this article, or claim that may be made by its manufacturer, is not guaranteed or endorsed by the publisher.

## Footnotes

^1^World Health Organization. https://covid19.who.int/

^2^World Health Organization. http://ClinicalTrials.gov
